# Uterine Hæmatocele

**Published:** 1862-01

**Authors:** Chas. West

**Affiliations:** London, England


### UTERINE HÆMATOCELE.

Reprinted from West on the *Diseases of Women.*

Within the past few years attention has been directed, chiefly by French writers, to cases in which *tumors have been formed* in the immediate vicinity of the uterus *by the effusion of blood* either into the cellular tissue around the womb, or into the peritoneal cavity in the *cul-de-sac* between the uterus and rectum. In both instances the hemorrhage is generally associated with some previous disorder of the menstrual function, often with its temporary suppression; the congestion of the sexual organs relieving itself by a profuse outpouring of blood, for which effusions the name of *uterine*, *retro-uterine*, or *peré-uterine hæmatocele* has been proposed.

When the hemorrhage takes place into the peritoneal cavity, its source has probably in the first instance been the lining membrane of the uterus itself and the Fallopian tubes, whence escaping at their fimbriated extremities it collects in the *cul-de-sac* behind the uterus. In one post-mortem examination this process was seen in actual course of occurrence, both tubes being distended with blood, and a partially decolorized coagulum hanging from the extremity of one of them. The blood thus poured out speedily excites inflammation, and adhesions forming between the adjacent coils of intestines shut it out from the cavity of the abdomen. It here undergoes within the artificial cyst that encloses it, the same changes as are incidental to sanguineous effusions elsewhere. Sometimes the blood is altogether removed by absorption, and adhesions between the uterus and adjacent viscera remain the only evidence of the bygone mischief. At other times an aperture of communication forms with the rectum, or more rarely with the vagina, and the decomposed blood is expelled, the patient either altogether recovering, or the sac remaining a pus-secreting surface, and pelvic abscess succeeding to the hæmatocele, as in a case which came under my own observation. In cases which have a fatal issue this is due either to the recurrence of hemorrhage exhausting the patient, or more commonly to the irritation extending beyond its original seat, and at length involving the whole of the peritoneum in a general inflammation. In two out of eight post-mortem examinations of which I have found a record, the hemorrhage seemed to have been furnished entirely from the uterus and Fallopian tubes; in one the vessels of the ovaries had given way under a more than usually intense congestion of those organs. In one it appeared to have had a two-fold source, being derived in part from the tubes, in part from the vessels of the broad ligament, into the tissues of which blood was effused. In two of the remaining four cases the blood was poured out behind the uterus but beneath the peritoneum; in one beneath the peritoneum in the iliac fossa, and in the fourth between the folds of the broad ligament.

We learn, then, from these observations the existence of a previously unknown hazard attendant on disorders of the sexual system in women: that not merely may intense congestion lead to profuse and dangerous floodings, or functional disturbance issue in inflammation of parts in the vicinity of the uterus, but also that vessels may give way, and hemorrhage take place inwardly, in situations where it is hard to discover, and still harder to suppress. As might be expected, the accident is one which takes place only during the period of sexual vigor, it having occurred in 21 women at the following ages:—

| | | | | | |
|---|---|---|---|---|---|
| Under | 20 | | | in | 2 |
| Between | 20 | and | 25 | " | 2 |
| " | 25 | " | 30 | " | 7 |
| " | 30 | " | 35 | " | 5 |
| " | 35 | " | 40 | " | 4 |
| At | | | 40 | " | 1 |
| | | | | | 21 |

Of the above 21 patients 15 were married, 3 were single, and the civil state of the other 3 is not mentioned.

The affection has scarcely been observed often enough or with sufficient minuteness to allow of its features being sketched with complete exactness, though in all the cases of it there is a sort of general family likeness which I think would enable the attentive observer usually to recognize it, or which at least would arouse his suspicions as to its possible character. Of the four cases that came under my own notice, one was that of an unmarried woman, aged twenty-two, who having long suffered from attacks of pain of a paroxysmal character in the left iliac region, was surprised at the age of nineteen by a profuse discharge of a dirty reddish brown color from the vagina, which continued in varying quantity for many weeks, and was then succeeded by a puriform discharge occurring in gushes, which continued down to the time of her coming under my care. A tumor in the iliac region, and another felt behind the uterus fixing that organ in its place, were the evidences of some bygone inflammation; of an old pelvic abscess in short, the origin of which, in an effusion of blood was rather inferred from the patient's previous history than actually demonstrated. Puncture of the abscess and the injection of a solution of iodine into its cavity were followed by its complete cure. In the other cases the accident was of recent occurrence, and its symptoms were sufficiently characteristic to remove all doubts as to its nature. The patients were married women of the respective ages of 33, 24 and 25 years. In the first, exertion on the second day after miscarriage at the sixth week was followed by great increase of the sanguineous discharge, which continued for twelve weeks. At the end of this time a vaginal examination detected a tumor behind the uterus of the size of an apple. On being punctured it gave issue to a reddish-brown discharge, the continuance of which for three weeks was followed by the complete disappearance of the swelling. In the second patient, who for five years had lived in sterile marriage, the symptoms gradually developed themselves during the persistence for two months of a discharge, supposed to be menstrual. Here, too, a tumor behind the womb gave issue, when punctured, to a black offensive discharge, which evidently consisted of decomposed blood, and the patient having surmounted an attack of peritonitis perfectly recovered. The third case so well illustrates the symptoms and the dangers of the affection, that it seems to me deserving of relation somewhat in detail.

A tall, stout, and tolerably healthy-looking woman, twenty-five years old, who had been married for seven years, had been pregnant four times, and had given birth to three living children, of whom the youngest was twelve months old, was admitted into St. Bartholomew's Hospital on February 22d,

1851. Her general health had been good, her labors had been natural, and after all of them she had menstruated regularly during the whole period of lactation. After her third labor matters went on as usual until Christmas, when she menstruated naturally, but ever since that time a sanguineous discharge, neither very profuse nor intermingled with coagula, had been constantly present. For a month she had had pain of a bearing-down character, aggravated by exertion, but not notably relieved by rest, nor by any particular position; and she had also for the same time suffered from occasional fainting fits. Micturition was frequent and painful, and her urine was reported to be both scanty and high-colored. A medical man whom she had consulted told her that "her womb was down."

The abdomen was large and somewhat tense, its enlargement being due to the presence of a tumor, the surface of which was slightly uneven, occupying the whole of the left side, extending three inches above the umbilicus, reaching about three inches across the mesial line, though gradually sloping downwards, so that on the right side its upper margin was an inch and a half below the umbilicus. The tumor was firm, non-fluctuating, very tender to the touch, especially in the left iliac region.

The finger on being introduced into the vagina came almost immediately on a somewhat firm, elastic tumor, of an oval shape, of about the thickness of the wrist, and which had pushed before it the posterior vaginal wall. This tumor seemed to pass over into the substance of the uterus, about half an inch behind its orifice, the whole organ being so misplaced that the os uteri was felt lying horizontally immediately behind the symphisis pubis. The finger passed up in the front and right side of the pelvis without encountering any resistance; but at the left side and posterior part of the pelvis a firm tumor was felt apparently continuous with that immediately behind the uterus. The vessels of the tumor pulsated very forcibly.

About three ounces of a bloody fluid were drawn off on the tumor being punctured with a grooved needle through the vagina. The microscope discovered nothing but blood-corpuscles in the fluid, and with the view of emptying the tumor if possible, and of thereby relieving the painful pressure on the rectum, which occasioned much distress, a Pouteau's trocar and canula were introduced, but only about four ounces of fluid of the same character as before were let out. The tumor was not much thereby diminished in size, nor was the patient's discomfort much alleviated. On February 27th, no fresh interference having been resorted to, she was seized with peritonitis, during the course of which there was manifest increase of the tumor, which extended more towards the right side of her abdomen. By the 3d of March all active symptoms were subdued, and on that day the patient passed two copious evacuations, which were perfectly black, and apparently consisted entirely of altered blood. The same afternoon, too, she experienced a sensation as of something giving way internally, and this was immediately followed by an abundant gush, from the vagina, of very fetid fluid, resembling coffee-grounds in appearance. This fluid flowed at first very abundantly, afterwards more scantily till morning, when it ceased, though another gush of it took place on the following day, and afterwards recurred occasionally for several days, acquiring by degrees a lighter color, and becoming at last a dirty sero-purulent matter. Very slowly the patient's general health improved, while at the same time her abdomen diminished in size, and having measured forty-six inches on her admission had shrunk to forty inches on March 25th. The tumor in the left hypogastric region at the same time manifestly diminished in size and became more mesial in its position; and on April 5th the uterss had nearly regained its natural situation; there was no longer any distinct tumor behind it, but a hard, semi-cartilaginous thickening, ill-defined as to its extent and relations. On April 17th all discharge from the vagina finally ceased, and on May 5th all trace of abdominal tumor has completely disappeared, the position of the uterus was quite natural, the thickening behind it was much lessened. A year afterwards I again saw the woman; she was in perfect health, menstruating regularly; there was no trace of abdominal tumor, the uterus was perfectly movable, and there was scarcely any thickening to be felt behind it, or to its left side.

In its main features this case corresponds very closely with the description of uterine hæmatocele given by M. Nêlaton and others. Though some form of the disorder of the menstrual flux usually precedes the attack, the suppression of the discharge does not seem to be so constant as might, on theoretical grounds, have been anticipated; for sometimes irregularity has been observed both in its return and in the quantity of blood lost; at other times actual menorrhagia, and at others again a flow of blood, not alarming in its quantity, but at length causing anxiety by its continuance.* In most cases, too, even though the menses had been previously suppressed, a somewhat profuse flow of blood, sometimes for a few days, sometimes for a few weeks, precedes the actual occurrence of the internal hemorrhage; but the development of the acute symptoms generally follows a temporary diminution or cessation of the sanguineous discharge. The acute symptoms scarcely ever appear till after the sanguineous discharge has either ceased completely, or has become much diminished in quantity. The symptoms are those of general febrile disturbance, seldom, however, very severe, accompanied by abdominal pain, and usually by enlargement of the abdomen. Even of their own accord, these febrile symptoms usually subside, and the pain also diminishes; a sense of weight in the pelvis, bearing down, difficult micturition, and still more difficult defecation remaining behind, and leading by the distress which they occasion to a vaginal examination, and to the discovery of the pelvic tumor.

When matters have reached this stage, the subsequent progress of the case seems to depend on circumstances. Puncture of the tumor may be followed by the complete evacuation of its contents, and the rapid recovery of the patient; or an expectant mode of treatment may be succeeded by the slow absorption of the blood, and by gradual convalescence. But events may follow a different course, and one far less auspicious: peritonitis may come on as the result perhaps of some fresh effusion of blood, or in the course of nature's efforts to eliminate it; and this peritonitis occurring in a patient already weakened by the hemorrhages may prove fatal. Or, after more or less suffering, the blood may find a passage by the bowel, or by the vagina, or as in the case just related, by both at once; and with its discharge the swelling may disappear, and the patient eventually regain perfect health; her whole illness having extended over a period of from two months to six or seven.

(*To be Continued.*)

---

Infant Mortality In Ireland.—Despite legislative enactments favoring the poorer classes, notwitstanding the absence of famine or pestilence, the population of Ireland has decreased by 787,842 souls, which amounts to a proportion of 12.02 per cent. in the decade of years.—*Lancet.*

* In 13 out of 26 cases, suppression of the menses, or the irregularity of their return, which was postponed beyond its proper time, preceded the development of the symptoms of the effusion; in 6, on the contrary, there was menorrhagia, or a constant sanguineous flow, and in one instance abortion was followed for two months by constant, though not profuse hemorrhage. In six of the cases, or in rather less than a fourth, pain preceded the acute symptoms, but neither suppression of the menses nor any other form of menstrual disorder.

